# Estimating HIV Incident Diagnoses Among Men Who Have Sex With Men Eligible for Pre-exposure Prophylaxis but Not Taking It: Protocol and Feasibility Assessment of Data Sources and Methods

**DOI:** 10.2196/42267

**Published:** 2023-03-07

**Authors:** Patrick Sean Sullivan, Eric Hall, Heather Bradley, Travis Sanchez, Cory R Woodyatt, Elizabeth S Russell

**Affiliations:** 1 Department of Epidemiology Rollins School of Public Health Emory University Atlanta, GA United States; 2 Department of Epidemiology School of Public Health Oregon Health Sciences University Portland, OR United States; 3 Epidemiology Merck & Co, Inc Rahway, NJ United States

**Keywords:** HIV incidence estimates, pre-exposure prophylaxis, commercial pharmacy data, HIV, HIV epidemic, surveillance data, public health, men who have sex with men, health monitoring, clinical trial design

## Abstract

**Background:**

HIV incidence estimates are published each year for all Ending the HIV Epidemic (EHE) counties, but they are not stratified by the demographic variables highly associated with risk of infection. Regularly updated estimates of HIV incident diagnoses available at local levels are required to monitor the epidemic in the United States over time and could contribute to background incidence rate estimates for alternative clinical trial designs for new HIV prevention products.

**Objective:**

We describe methods using existing, robust data sources within areas in the United States to reliably estimate longitudinal HIV incident diagnoses stratified by race and age categories among men who have sex with other men (MSM) eligible for pre-exposure prophylaxis (PrEP) but not taking it.

**Methods:**

This is a secondary analysis of existing data sources to develop new estimates of incident HIV diagnoses in MSM. We reviewed past methods used to estimate incident diagnoses and explored opportunities to improve these estimates. We will use existing surveillance data sources and population sizes of HIV PrEP-eligible MSM estimated from population-based data sources (eg, US Census data and pharmaceutical prescription databases) to develop metropolitan statistical area–level estimates of new HIV diagnoses among PrEP-eligible MSM. Required parameters are number of new diagnoses among MSM, estimates of MSM with an indication for PrEP, and prevalent PrEP use including median duration of use; these parameters will be stratified by jurisdiction and age group or race or ethnicity. Preliminary outputs will be available in 2023, and updated estimates will be produced annually thereafter.

**Results:**

Data to parameterize new HIV diagnoses among PrEP-eligible MSM are available with varying levels of public availability and timeliness. In early 2023, the most recent available data on new HIV diagnoses were from the 2020 HIV surveillance report, which reports 30,689 new HIV infections in 2020, and 24,724 of them occurred in an MSA with a population of ≥500,000. Updated estimates for PrEP coverage based on commercial pharmacy claims data through February 2023 will be generated. The rate of new HIV diagnoses among MSM can be estimated from new diagnoses within each demographic group (numerator) and the total person-time at risk of diagnosis for each group (denominator) by metropolitan statistical area and year. To estimate time at risk, the person-time of individuals on PrEP or person-time after incident HIV infection but before diagnosis should be removed from stratified population size estimates of the total number of person-years with indications for PrEP.

**Conclusions:**

Reliable, serial, cross-sectional estimates for rates of new HIV diagnoses for MSM with PrEP indications can serve as benchmark community estimates of failures of HIV prevention and opportunities to improve services and will support public health epidemic monitoring and alternative clinical trial designs.

**International Registered Report Identifier (IRRID):**

DERR1-10.2196/42267

## Introduction

There is an urgent need to develop additional options for HIV pre-exposure prophylaxis (PrEP) for men who have sex with men (MSM). MSM are disproportionately impacted by HIV in the United States and globally [1], and models in multiple countries have suggested that achieving high coverage of PrEP will be required to realize substantial reductions in HIV incidence [2-4]. The prevention value of PrEP has not been fully realized in the United States for several reasons [5]. First, levels of PrEP use in the United States are well below the levels of PrEP use that will be required to result in substantial reductions in HIV incidence [6]. Second, MSM are not monolithic, and future PrEP products should be developed to meet diverse prevention needs. For example, PrEP approaches that increase the flexibility of dosing regimens, minimize side effects, offer options for discretion, and offer multiple options that promote adherence and increase flexibility for men will help increase PrEP choices for diverse patient needs. Higher uptake, longer persistence, and higher adherence that can be realized through increased choices will increase the impact of PrEP on HIV incidence [2,7]. Third, although current levels of PrEP use are comparable in Black and White MSM [6], given the higher burden of new HIV infections that occur in Black MSM, equal use of PrEP is not equitable [8,9].

The US national Ending the HIV Epidemic (EHE) strategy is predicated on the need to concentrate resources for HIV prevention and treatment in a relatively small number of communities that are most highly affected by HIV. The plan identifies 57 priority jurisdictions that, together, account for 50% of new HIV infections in the United States [10]. Within these jurisdictions, there is considerable variability in HIV risk by behavioral risk, race or ethnicity, and age. Overall HIV risk varies across jurisdictions, but Black and Hispanic MSM have the highest HIV risk in nearly every geographic location [11]. To reach EHE goals for reductions in HIV incidence, effectiveness of prevention methods will need to be evaluated locally, and across key populations that vary by geography, age, and race or ethnicity. Currently, PrEP use relative to need is lower among Black versus white MSM, and this disparity may extend to, or even be exacerbated by, development of novel PrEP pharmaceutical products [6]. The availability of estimates of new HIV diagnoses over time—a proxy for HIV incidence—by geography, race, and age will help to monitor racial disparities in current and future PrEP use and demonstrate how improvements in adherence and the resulting effectiveness improve equity across populations.

We are now in a phase of increasing PrEP options beyond daily oral tablets and gluteal injections every 8 weeks [12-18]. Increased and equitable PrEP uptake will likely require the development of multiple formats for PrEP (eg, daily oral, event driven, monthly oral, and longer-acting injectable) that are congruent with users’ lives, access to care, and preferences for prevention [19,20]. A more robust pipeline of PrEP products offers opportunities to reduce barriers for more people and increase effective PrEP use, as has been demonstrated in the contraceptive field [21]. However, there are clinical trial design challenges to sustaining the progress toward developing more modes for PrEP in populations with diverse needs and preferences.

Given the proven options for PrEP, the traditional trial design for new PrEP medications would be a noninferiority trial [22,23], such as the recently reported trials of combination emtricitabine and tenofovir alafenamide [15] and long-acting cabotegravir [24], designed to demonstrate that HIV incidence in those taking the experimental agent was not higher than incidence in those taking a proven PrEP option. These types of trial designs rely on enough incident infections within the trial population to demonstrate efficacy of the investigational agent. Demonstrating efficacy with these designs is becoming more challenging in the field of HIV prevention, where proven products are highly efficacious in clinical trial settings (with few incident infections), requiring impractically large sample sizes to achieve statistical power for noninferiority margins [25]. There is an argument that if multiple highly effective products are available such that traditional trials are not feasible, then there is no need for new products. However, this would ignore the ongoing HIV incidence outside of these highly controlled settings [26]. The significant ongoing unmet need for HIV prevention and the inequity it represents require us to develop methods that allow for rigorous efficacy and safety trial data for new HIV prevention methods.

Alternative trial designs for future HIV prevention products have been the topic of many recent meetings [27-29] and publications [25,30-33]. One of these alternative approaches is to compare the incidence in those receiving the investigational medication to the incidence in a counterfactual group (ie, a hypothetical contemporary group of persons with similar risk characteristics who would be eligible for the PrEP treatment, but who were not taking PrEP).

Ideally, a counterfactual group that would be used as a comparison group to evaluate the performance of a novel PrEP agent should reflect what the HIV incidence would have been in a comparable group of PrEP-eligible MSM (ie, men who would be eligible for PrEP by the Centers for Disease Control and Prevention [CDC] clinical criteria, but who were not enrolled in the trial and were not taking PrEP outside of the trial). MSM who live in the study community, who would meet study eligibility criteria, and who could enroll in the trial but do not enroll constitute a potentially appropriate control group. The development of such a counterfactual control group, however, has important challenges. First, there is no enumerated registry of MSM who would have indications for PrEP. Second, unlike in a trial, where participants are regularly screened for HIV infection as an outcome, not all community-acquired cases of HIV infection will be diagnosed and documented. Third, the characteristics of men who do not enroll in the trial of the novel agent might be different in important ways from men who choose to enroll (eg, for understandable reasons such as mistrust of health research [34], Black and Hispanic people were historically less likely to enroll in HIV vaccine trials [35]).

Here, we propose a protocol to estimate the incident HIV diagnoses as a proxy of HIV incidence of HIV among PrEP-eligible MSM at the level of metropolitan statistical area (MSA). These estimates could represent a valuable source of data as a counterfactual reference group to evaluate the efficacy of PrEP in future trials of novel PrEP agents and would have other benefits to community planning and prevention efforts. To increase the rigor of this approach, we propose incorporating multiple population-based data sources, building on previously published methods [36] and incorporating methodologic improvements, including multiple stratifications of men by age and race to reflect important heterogeneities in HIV risks.

## Methods

### Modeling Equation

This surveillance study will be conducted using structured secondary data and will build upon the methodology outlined by Mera et al [36]. To estimate the new HIV diagnosis rate among MSM with PrEP indications, we will estimate the number of new diagnoses among this group (numerator) and the total person-time at risk of diagnosis for this group (denominator) by MSA and year. The approach is summarized by Equation 1 and described in detail below.




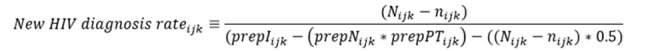




where *i* is demographic strata, *j* is MSA*,* *k* stands for year, *N* is number of new HIV diagnoses, *n* is number of new HIV diagnoses that are not in the MSM transmission category, *prepI* is *number of MSM with indication for PrEP*, *prepN* is *number of MSM PrEP users*, and *prepPT* stands for *average amount of time on PrEP my MSM in year k*.

### Numerator Data Sources

The numerator represents the annual number of new HIV diagnoses among MSM with an indication for PrEP, by MSA and demographic strata, and will use data from the annual CDC HIV surveillance reports [37,38]; these data are available through CDC AtlasPlus [39]. The surveillance data sets have been published using consistent methods since 2008 and are the largest and most representative data sources for HIV diagnoses in the United States. The HIV surveillance report includes the number of new HIV diagnoses reported per year to the National HIV Surveillance System stratified by transmission category (ie, MSM, people who inject drugs, and heterosexual), race or ethnicity (ie, American Indian or Alaska Native, Asian, Black or African American, Hispanic or Latino, Native Hawaiian or Other Pacific Islander, White, Multiple races), age (10-year increments), and residence. Additionally, the HIV surveillance report includes stratification by MSA, as defined by the Office of Management and Budget. As described by Mera et al [36], a cumulative total of all new HIV diagnoses can be adjusted by subtracting all new HIV diagnoses that are attributed to a transmission category that are not in the MSM transmission category.

### Denominator Data Sources

The denominator represents the total time at risk among MSM at risk of HIV acquisition. To estimate this for each stratum of interest, we will start with estimates of the number of adults that would be expected to have indications for PrEP [40] and remove person-time during which persons were on PrEP or acquired an incident HIV infection. Previous analysis of surveillance data has resulted in cross-sectional estimates of the number of adults in each HIV transmission risk category at the state level, who are then presumed to have an indication for PrEP [40]. To estimate the proportion of adults with a PrEP indication in each MSA by year, we will need to assume the proportion of persons with a PrEP indication is consistent within a jurisdiction and over time.

### Person-Time Calculations

The amount of person-time among PrEP users can be estimated with data on the total number of PrEP users within each jurisdiction and year and data on the average length of time that users were on PrEP during the calendar year. Large, national prescription data sets include information on the number of patients who received a prescription of emtricitabine/tenofovir disoproxil fumarate or emtricitabine/tenofovir alafenamide and corresponding diagnostic codes. Using a validated algorithm [41], we can exclude prescriptions that were made for other known indications, such as HIV treatment, postexposure prophylaxis, and chronic hepatitis B management. We will estimate the number of prevalent PrEP users by tabulating the number of persons that had at least one day of prescribed PrEP in the respective calendar year. AIDSVu.org [42] applies this algorithm to data from Symphony Health to report estimates of the total number of current PrEP users within each county [6]. The estimates from each central county within an MSA can be summed to calculate the number of current PrEP users within the MSA. Additionally, we will assume that the rate of new HIV diagnoses is constant throughout the calendar year. With this assumption, we expect, on average, that the amount of time at risk among persons who experienced a new HIV infection during the calendar year was 6 months (ie, half of a year).

### Ethical Approval

The proposed analyses will use deidentified and aggregated data sets; data about individuals are not provided in the data sets. The Emory University IRB has determined that this project does not require institutional review board review because it is not research with “human subjects,” nor is it a “clinical investigation” as defined in the federal regulations.

## Results

In early 2023, the most recent available surveillance data on new HIV diagnoses were from the 2020 HIV surveillance report, which was published in May 2022 [11]. This report indicates there were 30,689 new HIV infections in 2020, and 24,724 of them occurred in an MSA with a population of ≥500,000. Additionally, this report presents the number of new reported HIV diagnoses by MSA. The number of new HIV diagnoses, stratified by transmission category, race or ethnicity, or age, is typically published in supplemental volumes to primary HIV surveillance report, and the most recent supplementary volume with these data reflects diagnoses that occurred in 2018 [11].

Smith et al [40] developed a methodology for estimating the number of adults with PrEP indications by race or ethnicity and transmission category within each state. The most recent application of this methodology yielded estimates that indicate 1.1 million adults (813,970 MSM, 258,080 heterosexually active adults, and 72,510 persons who inject drugs) had indications for PrEP use in 2015. The methodology and data sources for these estimates can be expanded and updated. Additionally, assuming the proportion of persons with a PrEP indication is consistent within a state allows for the calculation of the total number of persons within each MSA (by transmission category and race or ethnicity) that have a PrEP indication.

AIDSVu.org annually releases new estimates of the current number of PrEP users, stratified by US county [42]. The methods for the production of these data have been previously reported [43]. The most recent estimates reflect 2019 and are further stratified by age group. We will use the publicly available county-level estimates from AIDSVu, which are updated annually; we will also explore further analyses of commercial pharmacy claims data to further stratify PrEP users within counties by age and race or ethnicity. Race or ethnicity data will be obtained from the matching of commercial pharmacy claims data to commercial credit reporting data, as has been previously reported by the CDC [40]; these matches will be evaluated for completeness and for potential biases related to missing data. Additionally, line-listed data from commercial pharmacy claims will be used to calculate the average duration of use for each demographic group of interest, using methods previously reported using claims data from large national pharmacy claims [44]. Our previous methods were focused on daily oral PrEP dosing strategies; we will develop and validate algorithms for identifying injectable cabotegravir [24] from claims data, to include this new PrEP agent and delivery route, and will continue to address new PrEP agents or dosing schedules as new agents become available (eg, monthly oral PrEP) [16].

## Discussion

### Principal Findings

We present an analytic approach for estimating population-level HIV incident diagnoses among PrEP-eligible MSM by jurisdiction, age group, and race or ethnicity. The proposed methods rely entirely on existing data and largely on publicly available surveillance data. This method would, for the first time, facilitate counterfactual estimates for the evaluation of novel PrEP pharmaceutical products by important characteristics of person, time, and place. HIV infections among MSM are highly concentrated among groups characterized by geographic location, age, and race or ethnicity, making it critical to evaluate new prevention methods within risk strata. Ensuring access to effective HIV prevention among populations with the highest risks for infection will require a nuanced understanding of how effectiveness of novel methods may vary across the demographic characteristics that determine HIV risk.

### Utility of New Data Produced

These estimates could be used in multiple ways to support alternative HIV prevention trial designs. They could serve as one estimate in building a background surrogate estimate for HIV incidence for an external control arm and could also be used in the context of noninferiority trials, the current standard for evaluating effectiveness of novel PrEP pharmaceutical products. These trials are costly and require large sample sizes to reach an adequate number of HIV infections among participants receiving prevention medications for comparison purposes. Dunn and Glidden [30] and Glidden et al [45] have proposed the ratio of averted infections in each trial arm as the summary measure for evaluating noninferiority of novel PrEP pharmaceutical products. This summary measure requires fewer assumptions about the noninferiority margin and a smaller sample size to evaluate noninferiority of the novel product than the commonly used ratio of HIV incidence in each trial arm. However, it requires an estimate of background HIV incidence for persons not taking either a proven PrEP product or the trial medication. Previously described approaches for estimating this counterfactual incidence parameter apply a Bayesian approach with estimates from historic research data on emtricitabine/tenofovir disoproxil fumarate effectiveness [32,45] or use the correlation between incidence of rectal gonorrhea and HIV to estimate the number of HIV infections that would have been observed in the absence of PrEP [46]. However, neither of these approaches makes use of population-based data, and they are therefore subject to bias to the extent that trial participants are dissimilar to persons among whom these assumptions are based.

The availability of estimates of PrEP incidence among PrEP-eligible MSM is also a critical building block to support the routine production of other data that will inform our understanding of the equitable use of PrEP and impact of PrEP. For example, we have described the PrEP-to-need ratio as an equity-based metric to depict the extent to which PrEP is used equitably by subpopulations (eg, Black and White MSM) [9]. These metrics have been calculated and reported at the state level [9], and the availability of data at the MSA level would allow the calculation of these metrics at levels relevant to local health jurisdictions. Our plan to produce stratified estimates by age will also allow the characterization and modeling of PrEP needs and exploration of PrEP prioritization strategies to maximize the impacts of PrEP in local communities.

### Enhancements to Existing Methods

The approach outlined here is based on previously published work by Mera et al [36], in which HIV diagnosis rates among PrEP-eligible individuals are estimated from 2012 to 2017. However, we propose several utility-added enhancements. First, use of the most current surveillance data will facilitate updated estimates for background HIV risk among PrEP-eligible MSM. Second, in addition to using updated surveillance data, the proposed method uses surveillance data that are stratified by age and race or ethnicity within jurisdictions. Stratification of key parameters in the surveillance data facilitates estimates specific to jurisdiction, age, and race or ethnicity. Third, previous estimates represent all PrEP-eligible individuals and assume adherence and persistence are constant across risk groups (eg, people who inject drugs, high-risk heterosexuals, and MSM). In reality, there may be important differences by risk group or age, and ignoring this heterogeneity might result in biased estimates of PrEP impact [47,48]. By focusing on MSM, who have the highest burden of HIV infection of all risk groups and the most unmet need for PrEP [9], our method will produce estimates that are directly informative to a trial counterfactual for MSM and to evaluating prevention needs of this important population. Last, we propose this method at the MSA level, but due to transparency of parameters used, it could be used at any geographic level for which needed data are available.

### Limitations

Despite these improvements, these methods have several continuing challenges. First, while we propose to use the most updated surveillance data for input parameters, surveillance data are typically published with a 1-2–year delay. If implemented for noninferiority trials, adjustments to HIV diagnoses based on historical reporting delays might be needed [49]. We acknowledge that this might be challenging in the coming 6-8 years because of disruptions in HIV diagnoses and reporting during the COVID-19 pandemic [50]. Moreover, modeled estimates needed for this work that rely on surveillance data inputs, such as the proportion of HIV-negative MSM with PrEP indications, require additional time to compute and publish after surveillance estimates are made publicly available. Population-based estimates of background HIV incidence will therefore not be perfectly contemporaneous to trial periods, due to reliance on publicly available data. Second, population-level HIV risk among PrEP-eligible MSM may be higher or lower than MSM enrolled in PrEP trials. To the extent that there is differential risk between trial participants and the background population, estimates of effectiveness of novel PrEP products may be subject to selection bias. However, the risk of selection bias is diminished substantially in the context of population-level estimation specific to jurisdiction, risk group, as well as age and race or ethnicity groups. Possibly the greatest limitation of population-based methods to estimate HIV incidence is that, currently, only HIV diagnosis data, rather than incidence estimates, are available within levels of needed stratifications. Therefore, the assumption must be made that diagnoses are roughly equal to incidence in a given year, and that this is constant across geography, age, and race or ethnicity. As HIV surveillance methods continue to improve, it is likely that incidence estimates will be available at finer levels of geography and demographic characteristics. Third, we recognize that the proportion of MSM with PrEP indications might not be stable over time. We will monitor the proportion of MSM with PrEP indications over time by using data from the American Men’s Internet Survey [6] and will adjust this parameter if the proportion of MSM with indications changes substantially over time. We will also conduct sensitivity analyses to this parameter in the first implementation of the protocol.

There are additional limitations and related assumptions that need to be considered to produce these estimates. For example, prescription data do not represent a full census of PrEP prescriptions, so PrEP use is underestimated in all MSAs and could be differentially underestimated. We assume that the average duration of PrEP use is the same for people who are and are not matched to race data; there are few data on PrEP persistence from observational studies stratified by race to inform the reasonableness of this assumption. We also assume that the proportion of MSM eligible for PrEP but not taking it is constant in MSAs over time and is consistent with state-level data. Most PrEP users in pharmacy data are men, and we assume that men on PrEP in pharmacy data are mostly MSM [43]. Prescription databases document the location of the provider who prescribed the medication, and not the patient; however, data suggest that people in urban areas usually reside 2-3 miles from their health care provider [51].

### Public Health Impact and Conclusions

Updates and improvements to some parameters outlined here would further increase the utility of these estimates. In particular, estimates for the population size of MSM in the United States and the proportion of HIV-negative MSM with PrEP indications should be updated with current data, and novel methods are needed to incorporate uncertainty around these estimates. Previous estimates of HIV incidence among PrEP-eligible individuals did not incorporate uncertainty from data sources derived from population samples [36]. Building uncertainty estimates into these 2 parameters would facilitate computation of standard errors around HIV incidence estimates that incorporate uncertainty across multiple data sources.

Population-based data on estimated incident HIV diagnoses among PrEP-eligible MSM at the MSA level are critical public health resources. Developing estimates of incident HIV diagnoses among PrEP-eligible MSM will provide an important data resource for use by local health departments to benchmark progress in HIV prevention programs. The use of local, meaningful indicators of HIV incidence and trends in incidence supports the essential functions of public health [52]. We plan to produce periodic updates to the estimates and make them publicly available to researchers and health departments through AIDSVu.org [42], as we have done with other data resources such as estimates of PrEP prescriptions among populations at the regional, state, and local geographic levels [9,43,53]. Estimates of incident HIV infections in PrEP-eligible MSM, in triangulation with other data sources, can inform assessments of local prevention coverage and opportunities.

## Abbreviations

CDC: Centers for Disease Control and Prevention
EHE: Ending the HIV Epidemic
MSA: metropolitan statistical area
MSM: men who have sex with other men
PrEP: pre-exposure prophylaxis

## Data Availability

Data used in the calculations will be available in aggregate form. Data on PrEP users is currently available through AIDSVu.org [54] for earlier years; updated public data sets will be posted as new data from this analysis are developed. Data on new HIV diagnoses will be taken from the CDC’s AtlasPlus website [55]; these data are also publicly available.
